# Highly NO_2_ sensitive caesium doped graphene oxide conductometric sensors

**DOI:** 10.3762/bjnano.5.120

**Published:** 2014-07-17

**Authors:** Carlo Piloto, Marco Notarianni, Mahnaz Shafiei, Elena Taran, Dilini Galpaya, Cheng Yan, Nunzio Motta

**Affiliations:** 1 Institute for Future Environments and School of Chemistry, Physics, and Mechanical Engineering, Queensland University of Technology, Brisbane, QLD 4001, Australia; 2 The University of Queensland, Australian Institute for Bioengineering and Nanotechnology, Australian National Fabrication Facility - QLD Node, Brisbane, QLD 4072, Australia

**Keywords:** caesium, conductometric, doping, drop casting, gas sensor, graphene oxide, highly sensitive, nitrogen dioxide

## Abstract

Here we report on the synthesis of caesium doped graphene oxide (GO-Cs) and its application to the development of a novel NO_2_ gas sensor. The GO, synthesized by oxidation of graphite through chemical treatment, was doped with Cs by thermal solid-state reaction. The samples, dispersed in DI water by sonication, have been drop-casted on standard interdigitated Pt electrodes. The response of both pristine and Cs doped GO to NO_2_ at room temperature is studied by varying the gas concentration. The developed GO-Cs sensor shows a higher response to NO_2_ than the pristine GO based sensor due to the oxygen functional groups. The detection limit measured with GO-Cs sensor is ≈90 ppb.

## Introduction

Graphene is a single layer of carbon atoms arranged in a honeycomb lattice [1–2]. Intrinsic low noise structure, large specific surface area and extraordinary mobility of carriers are the unique properties that make graphene-based materials excellent candidates for a wide variety of electrical applications [3]. One of the most promising applications is chemical sensing with detection limit down to ppb level [4–11]. Such ultrahigh sensitivity can play a crucial role in applications including health care, gas alarms, safety and environmental monitoring [12].

Theoretical [13–14] and experimental [15–19] studies have revealed that functionalization of graphene can improve significantly its gas sensing performance [20]. The presence of dopants or defects in the graphene lattice can increase the adsorption energy, i.e., the gas molecules can absorb more strongly on the doped or defective graphene than the pristine graphene resulting in an enhancement of the sensitivity or selectivity.

Recently, graphene oxide (GO), a graphene layer decorated with oxygen functional groups, has been subject to extensive research [8,21–24], as the synthesis of GO is the first step to easily obtain functionalized graphene [25]. GO can be synthesized from colloidal suspensions of graphite derivatives [26–29], e.g., graphite oxide, a method significantly cheaper and scalable than most of the common processes to make pristine graphene sheets, like chemical vapour deposition, epitaxial growth or mechanical exfoliation [30–33].

By dispersion and sonication of graphite oxide in aqueous solution or organic solvent, a colloidal suspension of GO sheets is produced. The density of oxygen functional groups can be easily controlled [28,34–38] making this process a good candidate for graphene functionalization. The oxygen groups of the resulting GO lead to the disruption of the graphitic structure, thus making the material electrically too much insulating for resistive gas sensing applications. However, the partial removal of oxygen groups, leading to reduced GO can be achieved by chemical [38–39], thermal [40–41] or ultraviolet-assisted process [42]. The conductivity and gas sensing performance of the reduced GO is comparable or superior to that of the pristine graphene [43], due to the oxygen defects that act as low energy adsorption sites.

To further enhance its gas sensing properties, reduced GO can be doped with alkali metals [18], similarly to what has been done in other carbon materials, to tune up the electronic properties for sensing applications [44].

Different research groups have reported high gas sensing performance of conductometric devices based on GO [25,35,45], reduced GO (rGO) [15,23–24,29,46–47] and functionalized rGO [18,48–50]. Prezioso et al. [25] have measured the NO_2_ sensing performance of GO drop casted on standard interdigitated Pt electrodes. They reported a very low detection limit (20 ppb), which is attributed to the high quality of their GO samples (large and highly oxidized flakes). Robinson et al. [46] demonstrated that by increasing the level of reduction it is possible to improve the response time and 1/*f* noise. It has also been proven by Yuan et al. [50] that reducing the thickness of the sensing layer below 5 nm results in a significant enhancement of the sensitivity [50]; although other authors claim that very thin layers would result in a not uniform conducting path [18]. The decoration of rGO with Pd nanoparticles using sputtering or by alternating current dielectrophoresis has shown an improvement in the sensitivity to NO by a factor of 5 (down to 2 ppb at room temperature) [15] as well as selectivity to hydrogen [18].

Increasing air pollution and global warming raised the demand for highly sensitive and portable NO_2_ gas sensors. To this purpose, metal oxide materials have been investigated reaching the lowest detection threshold of 0.1 ppm [51]. The high operating temperature of these devices, in the range of 200–400 °C, is a serious drawback that makes their utilization difficult in the field, where power consumption is a critical parameter. Carbon-based materials, such as graphene and chemically derived graphene, offer high sensitivity to cost ratio even when operating at room temperature [52].

In this article, we report for the first time the fabrication, characterization and gas sensing performance of a caesium-doped GO (GO-Cs) based conductometric sensor. Due to the reported catalytic activity of Cs, we believe that the sensing performance of the GO can be improved significantly [53]. Both pristine GO and Cs doped GO sensors have been tested towards different concentrations of NO_2_ gas at room temperature. The detection limit measured with GO-Cs sensor is ≈90 ppb.

## Experimental

### Device fabrication

GO materials were prepared by oxidation of graphite flakes following the method reported by Marcano et al. [54]. Commercially available graphite flake was purchased from Sigma-Aldrich. All other chemical used, (99.99% H_2_SO_4_, 85% H_3_PO_4_, 35% HCl, 30% H_2_O_2_, KMnO_4_) in this study were analytical grade and supplied by Sigma-Aldrich. Analytical grade ethanol, acetone and diethyl ether were used as solvents.

The graphite mixed with KMnO_4_ (ratio of 1:6) was combined with a mixture of H_2_SO_4_:H_3_PO_4_ (540:60 mL) acids. The reaction was stirred at 50 °C for 12 h. Subsequently, the resulting mixture, cooled at room temperature, was poured onto ice with 3 mL of 30% H_2_O_2_ and sifted through a 250 µm sieve. The filtrate was centrifuged at 4000 rpm for 30 min. The obtained material was washed with DI water, HCl and ethanol. After each wash, the mixture was sieved and centrifuged for 30 min at 4000 rpm. The final precipitate was coagulated with diehyl ether. Coagulated solid was dissolved in DI water and sonicated for 1 h. The resulting GO aqueous dispersion was cooled down for 24 h followed in a de-freezer and subsequently for 72 h in a freezer dryer at −51 °C under vacuum.

In order to synthesize GO doped with caesium (GO-Cs), the GO was diluted in water and mixed with Cs_2_CO_3_, following the method suggested by Liu et al. [55]. The obtained solution was stirred at room temperature for 30 min and sieved with a polyvinylidene fluoride membrane (0.2 μm). The precipitate was then added to water (30 mL) and filtered. The process was repeated twice to obtain dark solid GO-Cs.

Finally, the gas sensors were fabricated by drop casting of the prepared GO and GO-Cs materials onto 2 × 2 mm^2^ transducers and then they were placed in oven at 60 °C for 12 h. The transducers consisted of Pt interdigitated electrodes (IDT) (200 µm separation) deposited on 0.25 mm thick alumina substrates.

### Material characterisations

The structure and the composition of the synthesized GO and GO-Cs were analysed by field emission scanning electron microscopy (FESEM), X-ray photoelectron spectroscopy (XPS), atomic force microscopy (AFM), Raman spectroscopy and Kelvin probe force microscopy (KPFM).

XPS data were acquired using a Kratos Axis ULTRA X-ray photoelectron spectrometer incorporating a 165 mm hemispherical electron energy analyser. The incident radiation was monochromatic Al Kα X-rays (1486.6 eV) at 225 W (15 kV, 15 mA). Survey (wide) scans were taken at analyser pass energy of 160 eV and multiplex (narrow) high resolution scans at 20 eV. Survey scans were carried out over 1200–0 eV binding energy range with 1.0 eV steps and a dwell time of 100 ms.

Narrow high-resolution scans were run with 0.05 eV steps and 250 ms dwell time. Base pressure in the analysis chamber was kept at 1.0 × 10^−9^ Torr and during sample analysis 1.0 × 10^−8^ Torr. Peak fitting of the high-resolution data was also carried out using the CasaXPS software.

Raman spectroscopy was performed by using an ‘‘inVia Renishaw Raman microscope’’ with λ = 532 nm operated at 35 mW, with a 1 μm spot size, to investigate bond changes and defects in the material.

The KPFM was performed with a commercial AFM (Cypher-Asylum Research) equipped with an air temperature controller (ATC). The ATC flows temperature regulated, HEPA (High-Efficiency Particulate Absorption) filtered air through the Cypher enclosure. Closed-loop temperature control isolates the AFM from room temperature variations, minimizing thermal drift for imaging. During measurements the temperature was kept constant at 26 °C.

For all KPFM data shown here, we used conductive (Pt coated) AFM probes (NSG03 model from NT-MDT) with a nominal resonant frequency between 50 and 150 kHz. The GO and GO-Cs samples were deposited on gold-coated mica substrates from a liquid suspension (5 μg/mL). The Kelvin voltage was maintained with an integral gain of 4, no proportional gain, and an AC-voltage applied to the tip of 3 V.

### Gas sensing measurements

The GO and GO-Cs sensors response to NO_2_ was evaluated using a high precision multi-channel gas testing system, including a 1100 cc volume test chamber capable of testing four sensors in parallel, 8 high precision mass flow controllers (MKS 1479A) to regulate the gas mixture, 8 channel MFC processing unit (MKS 647C), a picoammeter (Keithley 6487) and a climatic chamber to control the temperature. The measurements were performed at room temperature with a mixture of synthetic air and NO_2_ gas in different concentrations (up to a maximum of 12.2 ppm of NO_2_ balanced in synthetic air). The right concentration of NO_2_ gas in air was obtained by adjusting the respective flow rates via the MFCs, while maintaining a total constant flow rate of 200 SCCM (mL/min). The response upon gas exposure was evaluated by measuring the sensors resistance variation with bias voltage of 3 V.

## Results and Discussion

### Material characterisations

**Figure 1:**
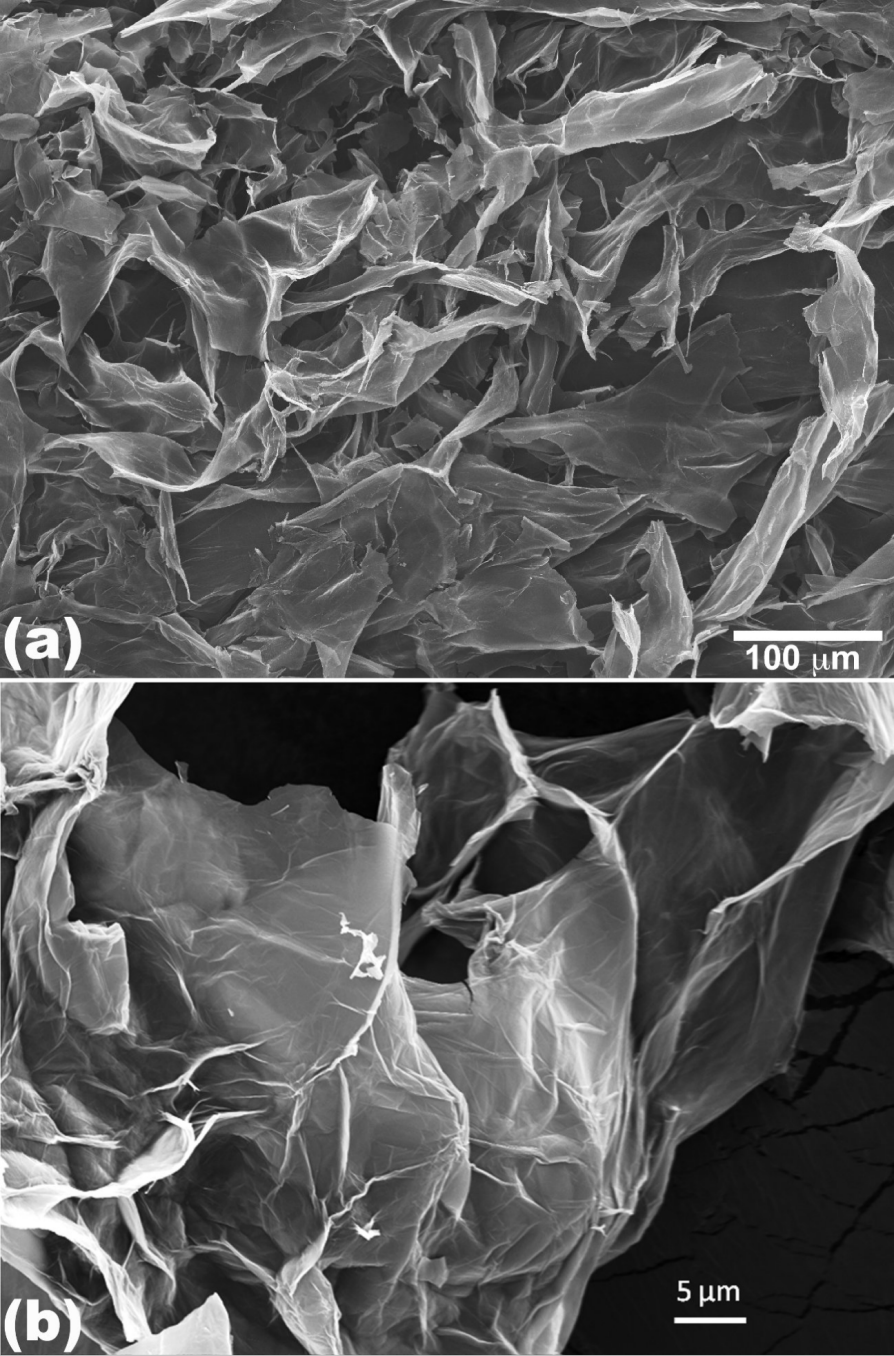
(a) Low magnification and (b) high magnification SEM images of graphite oxide flakes.

The morphology of the synthesized graphite oxide powder was investigated by FESEM (Figure 1). It is evident that the thin and aggregated flakes are stacked to each other with lateral sizes ranging from several hundred nanometers to several microns.

**Figure 2:**
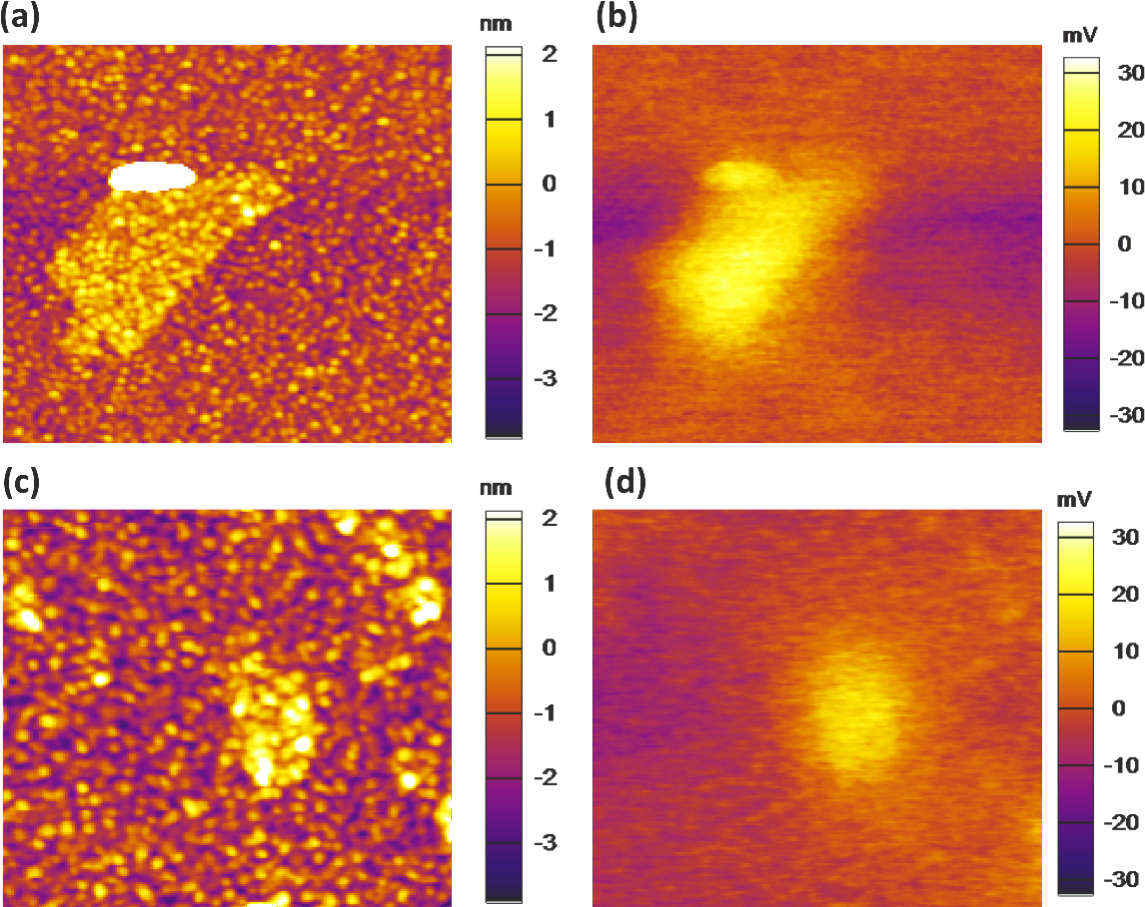
AFM and KPFM images of (a) and (b) a GO flake (2 × 2 μm); (c) and (d) a GO-Cs flake (1.4 × 1.4 μm).

AFM images (Figure 2a and 2c) confirm that most of GO and GO-Cs flakes are approx. 1 nm thick, corresponding to one monolayer, with a lateral size in the range of hundred nanometers [56–57]. The thickness of each GO layer is usually higher than the pristine graphene sheet because of the orthogonally bonded oxygen groups coming out from the surface [28,57–58].

Electrical characterizations were carried out with KPFM providing a potential map of the samples with a resolution of few mV. Figure 2b and 2d show a comparison of pristine GO and GO-Cs KPFM signals.

The measurements collected from several samples and on different flakes show a net difference in the potential map of GO (see Figure 2b) and GO-Cs flakes (see Figure 2d), with a drop of the average potential on a flake from 30 ± 3 mV in the GO to 19 ± 3 mV in the GO-Cs. We attribute this drop to the chemical reduction of the GO caused by the Cs_2_CO_3_ that tends to decrease the work function as observed by [55,59–61]. This result suggests that doped GO may have good performance as a gas sensing material.

**Figure 3:**
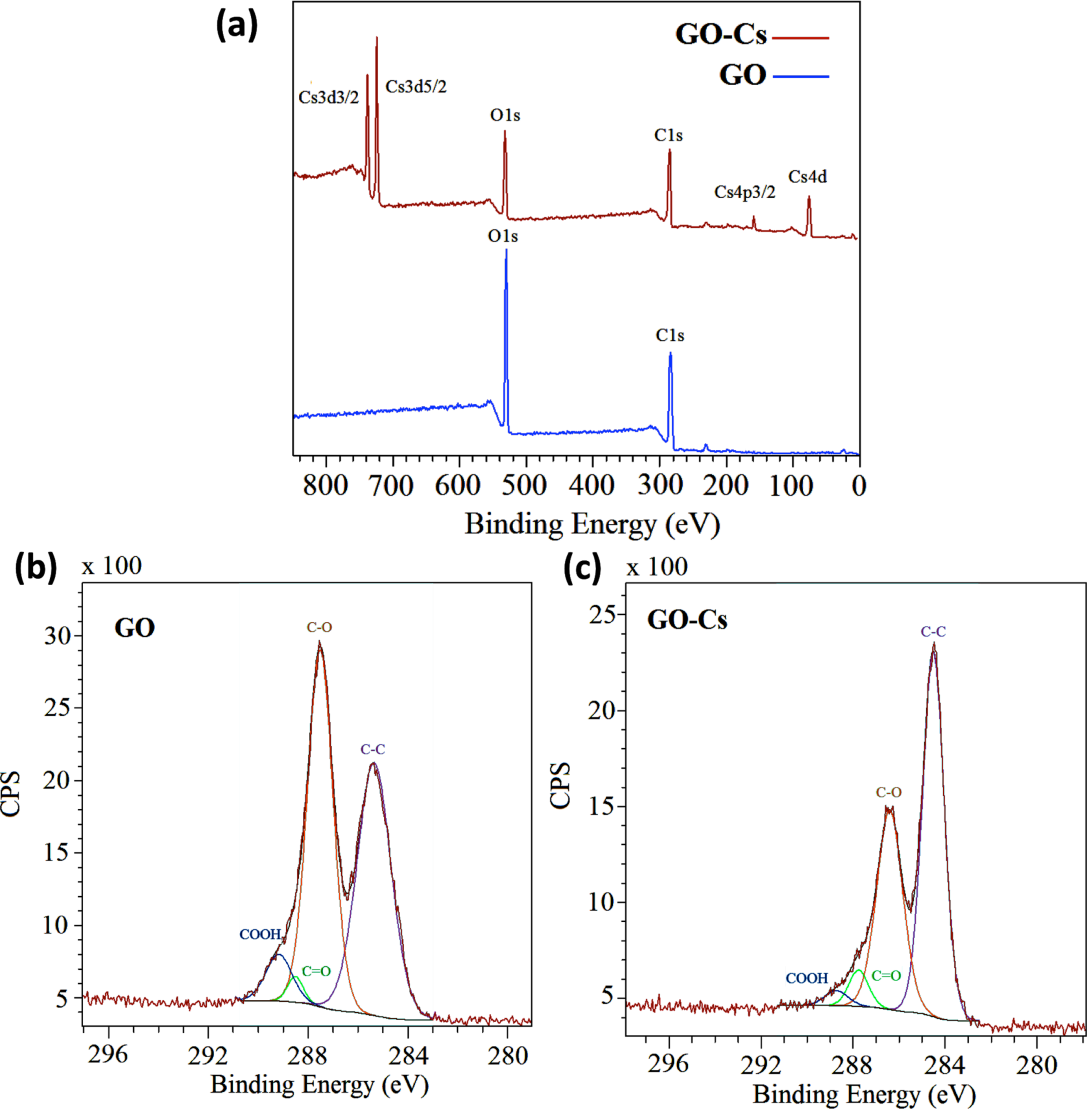
(a) XPS survey spectrum of GO (blue line) and GO-Cs (red line); High resolution XPS C1s spectra of (b) GO and (c) GO-Cs. The ≈1 eV shift towards lower binding energy of the peaks in (c) is due to the shift of the Fermi level caused by the doping.

**Figure 4:**
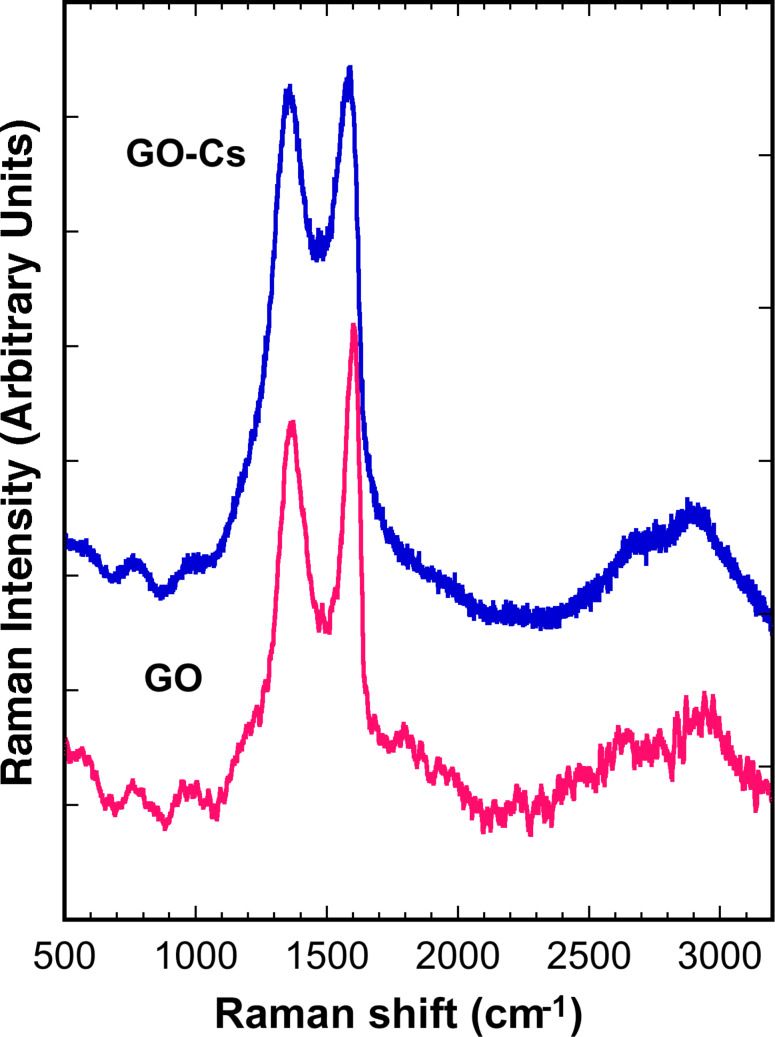
Raman spectra of GO-Cs and GO, displaying intense D and G peaks at **≈**1380 and **≈**1600 cm^−1^, respectively. The increase of the D peak, which is the signature of defects in graphene and GO, is clearly linked to the presence of Cs in GO-Cs.

XPS survey analysis of the GO (Figure 3a, blue line) confirms that the GO does not contain any contaminants and is largely oxidised with an oxygen content of ≈32%. A reduction of the oxygen content down to ≈24% is observed in the GO-Cs (Figure 3a, red line) survey spectrum, which confirm the presence of ≈5% Cs. In the high resolution XPS spectra of the C peaks (Figure 3b and 3c), we identify the C–C contribution as the peak at 285.3 eV binding energy, while the C–O, C=O and COOH groups are assigned to binding energies of 287.5, 288.4 and 289.1 eV, respectively [62–63]. Figure 3b and 3c show that the intensity of the C–O band in the GO-Cs decreases compared to the C–O band of the GO, confirming a reduction mechanism occurring in the GO due to the Cs_2_O_3_. Also the COOH peak decreases appreciably in the GO-Cs because of the substitution occurring between –COOH (that are usually at the periphery in the GO flakes), with –COOCs groups [55]. During the reaction, Cs^+^ is in fact expected to replace the H^+^ ions in COOH groups due to its higher reactivity. This is confirmed by the position of the Cs 3d5/2 peak at 724.1 eV (high resolution data, not shown), corresponding to the value of Cs bound to a carboxylic group [55]. It is worth also to notice the effect of the doping on the Fermi level, causing a 1 eV shift towards lower binding energy of all C peaks in the XPS spectra of GO-Cs (Figure 3c). The edge functionalization with the introduction of Cs^+^ does not cause much change in the carbon skeletons of the graphene oxide as observed by Liu et al. [55] and confirmed by our Raman spectra of GO and GO-Cs (Figure 4), where no appreciable shift is found in the D and G peaks. The 2D peak does not change as well, while the shape is compatible with the presence of several layers in the GO flakes. However a net increase in the D peak at 1360 cm^−1^ of the GO-Cs sample is a signature of the increased number of defects due to the presence of Cs^+^.

### Gas sensing performance

The GO-Cs and pure GO sensors were tested towards different concentrations of NO_2_ gas balanced in synthetic air at room temperature. The sensors response (*R*) was calculated according to the equation:


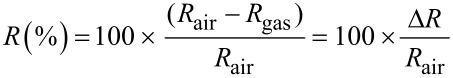


where *R*_air_ is the sensing film resistance under synthetic air only and *R*_gas_ is the film resistance during NO_2_ exposure.

As expected, the GO film showed a much higher resistivity than the GO-Cs film in the presence of air (10^13^ Ω vs 10^10^ Ω). The lower baseline resistance can be attributed to the reduction of oxygen groups in GO-Cs film as confirmed by XPS analysis (Figure 3). Being the value close to the resolution capability of our source meter (10 fA), the measurements of the GO film was affected by electrical noise.

**Figure 5:**
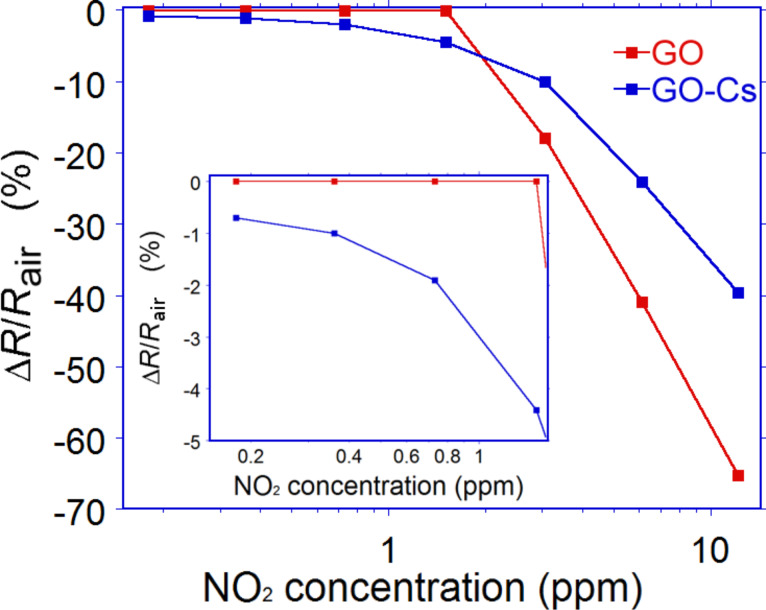
Response of the GO and GO-Cs based sensors as a function of NO_2_ concentration. The inset shows the response at very low concentrations.

We studied the response at room temperature towards different concentrations of NO_2_, ranging from 0.090 to 12.2 ppm. Both sensors exhibited a reduction in resistivity upon exposure to the gas, in agreement with the theory developed by Tang and Cao [14]: a negative charge is transferred to the NO_2_ molecules, mostly in correspondence of oxygen functional group, resulting in a p-type behaviour, which was also observed by Prezioso et al. [25]. For NO_2_ concentrations higher than 3 ppm both GO and GO-Cs exhibited a significant response, while at low concentrations the GO-Cs performed better. The GO-Cs sensor exhibited a significant response to NO_2_, down to concentrations as low as ≈91 ppb, while GO sensor did not show any response to concentrations below 3 ppm. This sensitivity enhancement could be attributed to defects introduced into the GO-Cs films during the doping process. Figure 5 shows the plot of the GO and GO-Cs sensors response as a function of NO_2_ concentration. Both responses are approximately linear and proportional to the gas concentration. Since NO_2_ is an oxidative gas with strong electron-withdrawing ability, the decrease in resistance confirms the p-type semiconductor behaviour of the sensors, like the one observed for carbon nanotubes [64]. For GO-Cs sensor a relative increase in the response (*R*_GO-Cs_) of 0.7, 1, 2, 4.4, 10, 24 and 40% was recorded for 0.18, 0.36, 0.73, 1.5, 3, 6.1 and 12.2 ppm NO_2_, respectively. Even at very low gas concentrations, a slope of about 3% ppm can be observed (inset of Figure 5), confirming that the as-prepared GO-Cs sample is highly sensitive to NO_2_. On the contrary, no appreciable response has been recorded for GO sensor in the presence of concentrations below 3 ppm, while a relative increase in the response (*R*_GO_) of 18, 41, 65% was recorded for 3, 6.1 and 12.2 ppm NO_2_, respectively. Table 1 summarises the response of the GO and GO-Cs sensors for comparison.

**Table 1:** Comparison of the GO and GO-Cs response towards NO_2_ with different concentrations.

NO_2_ [ppm]	*R*_GO_ [%]	*R*_GO-Cs_ [%]

0.18	—	0.7
0.36	—	1
0.73	—	2
1.5	—	4.4
3	18	10
6.1	41	24
12.2	65	39.6

We observed that the resistance of both sensors kept on decreasing even after 20 min exposure to NO_2_, reaching very slowly the saturation state. From deep saturation, the film required a very long time exposure to dry air to recover its original value. However, the significant variation of the resistance during the first phase of exposure can ensure a successful employment on the field of the sensing device. Therefore, we consider the exposure of approximately 4 min as an effective response time. This value has been chosen also in consideration of the time required to fill the volume of the gas chamber (1100 cc) with the target gas, which affects the dynamic response. The dynamic responses of GO and GO-Cs upon 4 min exposure to NO_2_ concentrations decreasing from 12.2 to 1.5 ppm have been measured simultaneously.

**Figure 6:**
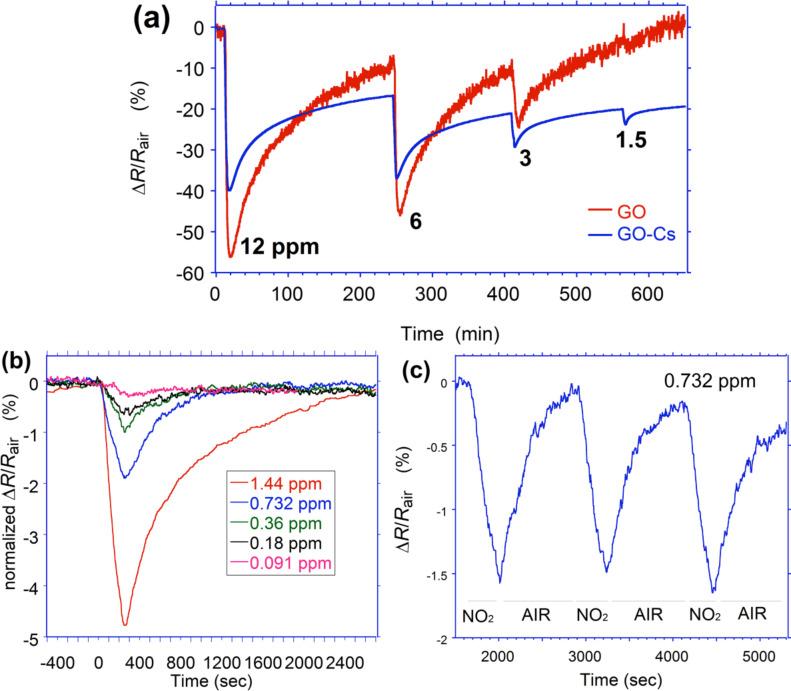
Response of (a) GO-Cs and GO based sensors towards NO_2_ with concentrations higher than 1 ppm; (b) GO-Cs based gas sensor after exposure to different concentrations of NO_2_ ranging from 0.091 to 1.44 ppm and (c) GO-Cs based sensor during 3 successive cycles of exposure to 0.732 ppm NO_2_ for 4 min and to dry air for 15 min.

As it can be seen from Figure 6a, the GO response is initially higher than GO-Cs, but decreases more rapidly. When exposed to 1.5 ppm, GO response is not anymore appreciable while a GO-Cs reaction is still evident. The noisier curves of GO is due to its higher resistivity value. Both sensors exhibit a long time to recover their initial value. Approximately 220 min are needed, although this value may be affected by the presence of residual NO_2_ molecules present in the 1100 cc gas chamber. While GO sensor recovers faster, GO-Cs is not able to fully recover its initial baseline. The dynamic response of the GO-Cs sensor upon 4 min exposure to 0.091, 0.18, 0.36, 0.732 and 1.44 ppm NO_2_ are shown in Figure 6b. The GO-Cs sensor reacts after few tens of seconds to the NO_2_ even at very low concentrations, down to 180 ppb. In terms of recovery time, for concentrations below 1 ppm, few minute exposures to dry air is enough to restore the original resistivity value. For higher concentrations, the recovery is longer, suggesting that the amount of Cs doping can be optimized to make a balance between the sensitivity and recovery time. This is in agreement with what observed by other researchers [25,46]. As shown in Figure 6c, the GO-Cs sensor exhibits a good repeatability, even if a slight drift in the baseline is observed. This may be due to the presence of gas molecules not yet desorbed from the sensor surface. An average time of 540 s is needed to recover after 240 s exposure to 0.732 ppm of NO_2_.

## Conclusion

We successfully fabricated and studied for the first time an NO_2_ sensor based on caesium-doped graphene oxide (GO-Cs). We demonstrated that caesium doping is an effective technique to reduce the GO, making it a promising material for gas sensing applications. XPS, Raman and KPFM results confirm the successful incorporation of Cs into the GO resulting in the reduction of oxygen groups. The developed GO-Cs based conductometric sensor exhibits a very low detection limit for NO_2_ (down to ≈90 ppb) at room temperature. This can be attributed to the p-character of the GO film, due to the intercalation of Cs atoms leading to the reduction of oxygen groups. However, the sensor shows very long recovery, making GO-Cs a good candidate for applications requiring high sensitivities, but not fast response. Future works will focus on investigating the effect of different species and concentration of dopants on improving the selectivity, response and recovery time.
